# Assessment of Double Outlet Right Ventricle Associated with Multiple Malformations in Pediatric Patients Using Retrospective ECG-Gated Dual-Source Computed Tomography

**DOI:** 10.1371/journal.pone.0130987

**Published:** 2015-06-26

**Authors:** Ke Shi, Zhi-gang Yang, Jing Chen, Ge Zhang, Hua-yan Xu, Ying-kun Guo

**Affiliations:** 1 Department of Radiology, West China Hospital, Sichuan University, 37# Guo Xue Xiang, Chengdu, Sichuan 610041, China; 2 National Key Laboratory of Biotherapy, West China Hospital, Sichuan University, 17# Section 3 South Renmin Road, Chengdu, Sichuan 610041, China; 3 Department of Radiology, West China Second University Hospital, Sichuan University, 20# Section 3 South Renmin Road, Chengdu, Sichuan 610041, China; Northwestern University, UNITED STATES

## Abstract

**Purpose:**

To evaluate the feasibility and diagnostic accuracy of retrospective electrocardiographically (ECG)-gated dual-source computed tomography (DSCT) for the assessment of double outlet right ventricle (DORV) and associated multiple malformations in pediatric patients.

**Materials and Methods:**

Forty-seven patients <10 years of age with DORV underwent retrospective ECG-gated DSCT. The location of the ventricular septal defect (VSD), alignment of the two great arteries, and associated malformations were assessed. The feasibility of retrospective ECG-gated DSCT in pediatric patients was assessed, the image quality of DSCT and the agreement of the diagnosis of associated malformations between DSCT and transthoracic echocardiography (TTE) were evaluated, the diagnostic accuracies of DSCT and TTE were referred to surgical results, and the effective doses were calculated.

**Results:**

Apart from DORV, 109 associated malformations were confirmed postoperatively. There was excellent agreement (κ = 0.90) for the diagnosis of associated malformations between DSCT and TTE. However, DSCT was superior to TTE in demonstrating paracardiac anomalies (sensitivity, coronary artery anomalies: 100% vs. 80.00%, anomalies of great vessels: 100% vs. 88.57%, separate thoracic and abdominal anomalies: 100% vs. 76.92%, respectively). Combined with TTE, DSCT can achieve excellent diagnostic performance in intracardiac anomalies (sensitivity, 91.30% vs. 100%). The mean image quality score was 3.70 ± 0.46 (κ = 0.76). The estimated mean effective dose was < 1 mSv (0.88 ± 0.34 mSv).

**Conclusions:**

Retrospective ECG-gated DSCT is a better diagnostic tool than TTE for pediatric patients with complex congenital heart disease such as DORV. Combined with TTE, it may reduce or even obviate the use of invasive cardiac catheterization, and thus expose the patients to a much lower radiation dose.

## Introduction

Double outlet right ventricle (DORV) is a complex congenital heart disease that occurs via embryological ventriculoarterial discordance such that the two great arteries completely or predominately arise (>50%) from the right ventricle [1]. Digital subtraction angiography is considered to be the gold standard initial imaging modality for application in cases of pediatric patients with DORV. However, it is an invasive procedure that carries risks associated with catheter-related complications. Another inevitable issue is its high radiation dose [2]. For the last decade, transthoracic echocardiography (TTE) has been regarded as the first-line imaging modality for pediatric patients with DORV. However, the small acoustic window and operator dependence are inherent disadvantages that affect its diagnostic performance. In addition, magnetic resonance imaging is a promising imaging modality because of its radiation-free nature. Nevertheless, some pediatric patients, particularly those with claustrophobia, are not suited for this examination because of the long acquisition time. Moreover, the need for high spatial resolution and elimination of motion artifacts to achieve optimal diagnostic image quality remains a current challenge [3].

Electrocardiographically (ECG)-gated dual-source computed tomography (DSCT) with high temporal resolution, low radiation doses, and excellent image quality has evolved into a reliable tool for pediatric patients with congenital heart diseases such as DORV [4]. Currently, the use of prospective ECG-gated scanning mode is preferred in pediatric patients with regular and low heart rhythm [5]. However, most pediatric patients have the relatively high or irregular heart rhythm, for these patients, it cannot achieve satisfactory image quality, particularly of intracardiac structures and coronary arteries [6]. However, the retrospective ECG-gated scanning mode, which allows a retrospective selection of the optimal instant for image reconstruction, can provide improved image quality for these patients [5, 7, 8]. To date, few studies have focused on the assessment of retrospective ECG-gated DSCT in pediatric patients who suffer from DORV. Thus, the purpose of this study was to evaluate the feasibility and diagnostic accuracy of retrospective ECG-gated DSCT for DORV and associated multiple malformations in pediatric patients.

## Materials and Methods

### Ethics statement

For its retrospective characteristic of this study, a application for exemption of patients’ informed consents was approved by the Institutional Review Board (IRB), and we pledged to abide by the declaration of Helsinki (2000 EDITION) in accordance with the relevant medical research rules of China in the study. In addition, no intervention was given in participations with strict secrecy for personal information and privacy. IRB at West China Hospital of Sichuan University approved this study.

### Study population

A total of 56 patients with DORV who were referred to our hospital between January 2010 and July 2014 were enrolled. The study inclusion criteria were patients <10 years of age and preoperative DSCT and TTE examination. The exclusion criteria were nonsurgical patients and unstable clinical conditions (n = 9). Finally, 47 patients remained (26 males, 21 females; mean age: 3.38 ± 3.11 years; range: 1.8 months to 10 years; heart rate: 136.8 ± 14.7 bpm). Written informed consent, including adverse reactions to the iodinated contrast agent and radiation exposure, was obtained from the guardians of the patients.

### DSCT

All examinations were performed using a DSCT scanner (Somatom Definition; Siemens Medical Solutions, Forchheim, Germany). Short-term sedation was achieved by an intravenous injection of chloral hydrate (concentration: 10%, 0.5 ml/kg) to the patients <6 years of age. The rest of the patients were asked to hold their breath during the scanning. The scanning was performed in the craniocaudal direction from the inlet of the thorax to 2 cm below the diaphragm level. A nonionic contrast agent (iopamidol, 370 mg/ml, Bracco, Sine Pharmaceutical Corp. Ltd., Shanghai China) was injected into an antecubital vein at a rate of 1.2–2.5 ml/s, followed by 20 ml of saline solution. The injected volume was adjusted to the body weight (1.5 ml/kg). Bolus tracking was used in a region of interest (ROI) in the descending aorta (Ao) with a predefined threshold of 100 HU. Image acquisition was triggered following a delay of 5 s when the ROI attenuation threshold reached 100 HU. Scanning was performed using a retrospective ECG-gated protocol with the following acquisition parameters: tube voltage, 80 kV; tube current, 100 mAs; gantry rotation time, 0.28 s; and pitch, 0.2–0.5 (selected according to heart rate; a higher pitch was used for higher heart rates). The ECG-pulsing window was set on Auto.

All acquired data were processed on a workstation (Syngo; Siemens Medical System, Forchheim, Germany). A slice thickness of 0.75 mm and an increment of 0.7 mm were chosen for image reconstruction. The multiplanar reformation (MPR), maximum intensity projection (MIP), and volume rendering (VR) were used for image analysis.

### Image analysis

Two experienced radiologists analyzed each subject in a blinded fashion. They analyzed and recorded the location of the ventricular septal defect (VSD), alignment of the two great arteries, and associated malformations using a sequential segmental approach. Disagreement was negotiated before the final diagnosis was given.

**Fig 1:**
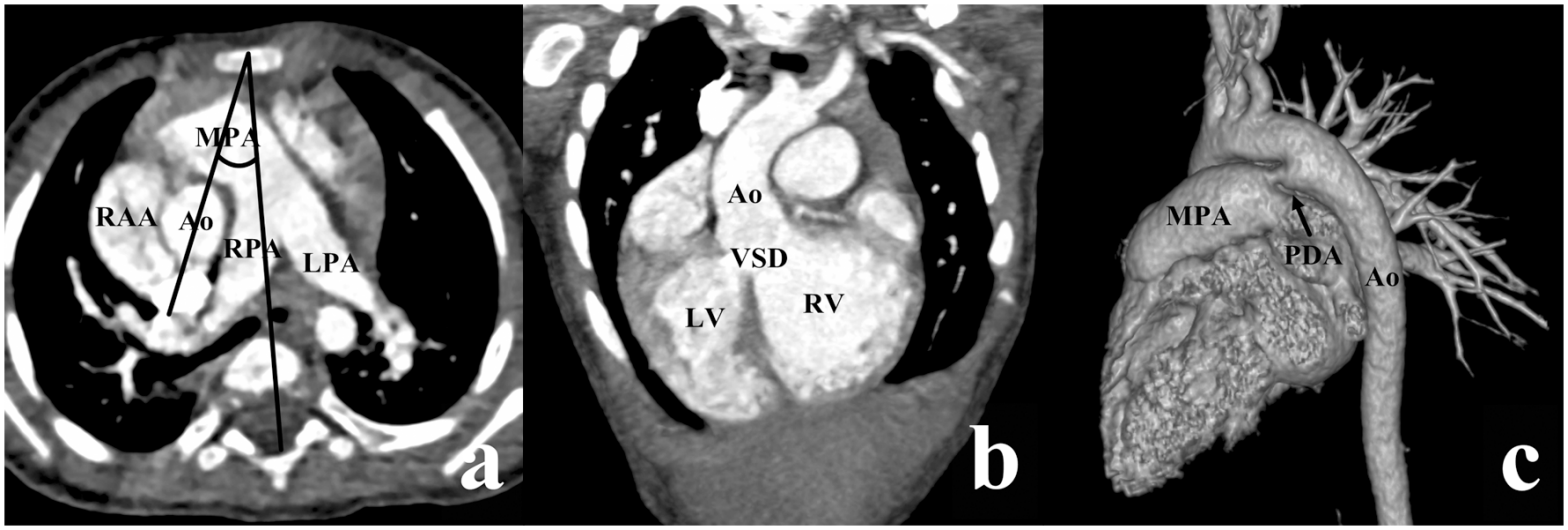
DORV with subaortic VSD in a male aged 9 months. (a) The aorta (Ao) was posterior and to the right of the main pulmonary artery (MPA) (the measured angle = 24°). (b) The VSD was situated below the aortic valve. (c) A ductus arteriosus (arrow) extended from the aortic arch to the MPA. LPA, left pulmonary artery; LV, left ventricle; RAA, right auricular appendage; RPA, right pulmonary artery; RV, right ventricle; PDA, Patent ductus arteriosus.

**Fig 2:**
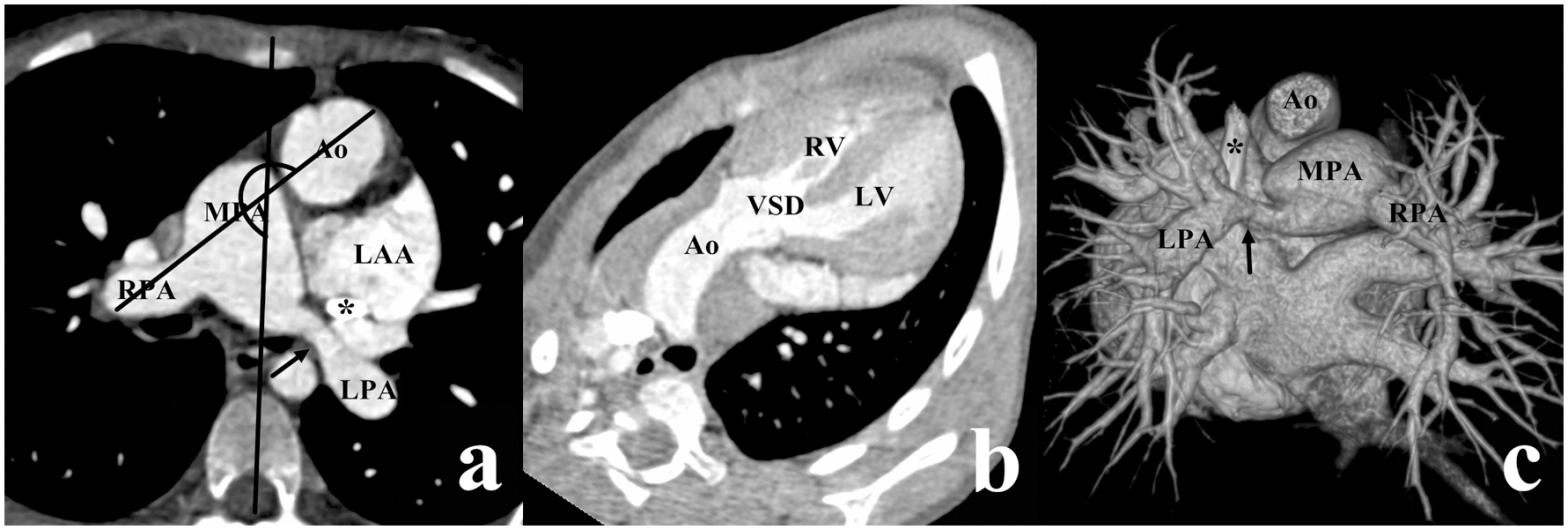
DORV with subaortic VSD in a female aged 6 years. (a) The aorta (Ao) was anterior and to the left of the main pulmonary artery (MPA) (the measured angle = 241°). (b) The VSD was situated below the aortic valve. (c) The volume rendering (VP) image showed the left pulmonary artery (LPA) stenosis (arrow) and the persistent left superior vena cava (star), as shown in Fig 2a. LAA, left auricular appendage; LV, left ventricle; RPA, right pulmonary artery; RV, right ventricle.

**Fig 3:**
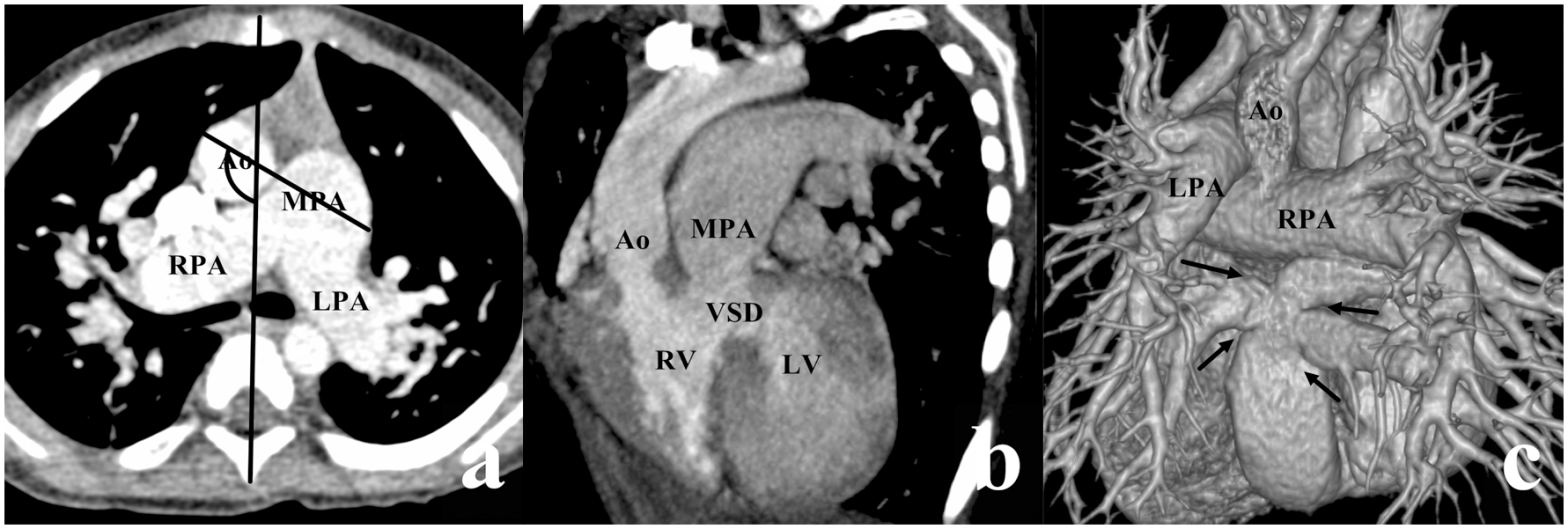
DORV with subpulmonary VSD in a female aged 3 years. (a) The aorta (Ao) was anterior and to the right of the main pulmonary artery (MPA) (the measured angle = 120°). (b) The VSD was situated below the pulmonary valve. (c) The volume rendering (VP) image showed the total anomalous pulmonary venous connection of the intracardiac type with 4 pulmonary veins (arrows) draining to the coronary sinus. LPA, left pulmonary artery; LV, left ventricle; RPA, right pulmonary artery; RV, right ventricle.

**Fig 4:**
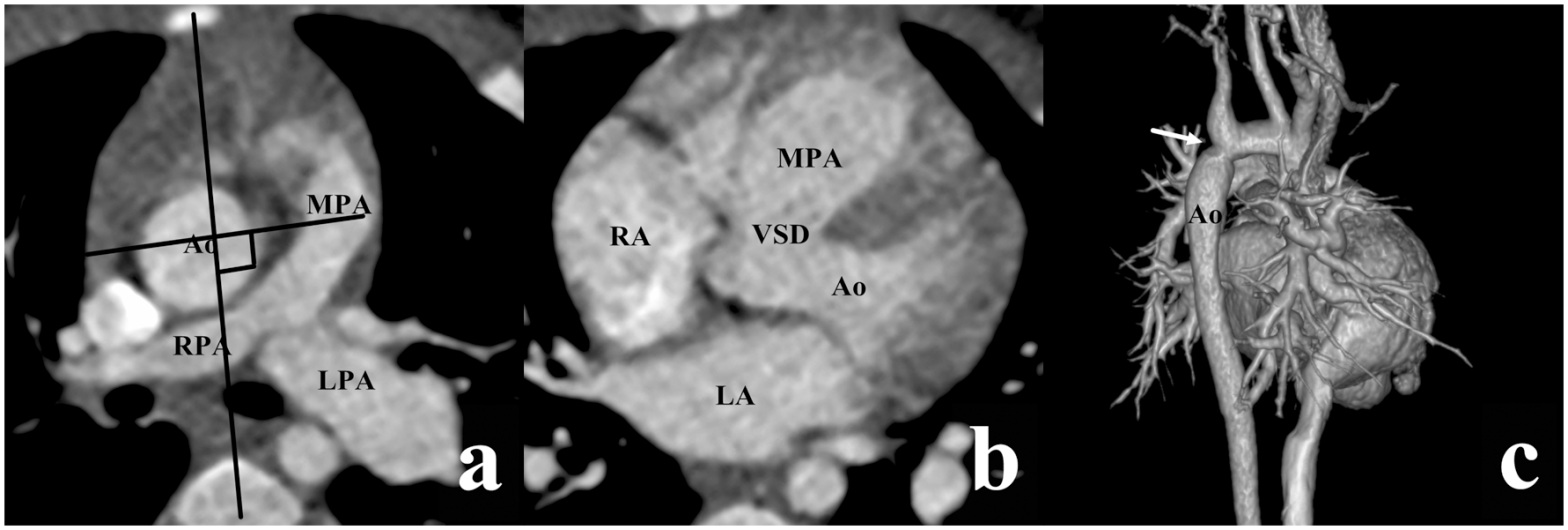
DORV with double-committed VSD in a female aged 3 months. (a) The two great arteries were side-by-side (the measured angle = 90°). (b) The VSD was situated below both the two arterial valves. (c) The volume rendering (VP) image showed the coarctation of the aorta (arrow) with poststenotic dilatation. Ao, aorta; LA, left atrium; MPA, main pulmonary artery; RA, right atrium; RPA, right pulmonary artery.

**Fig 5:**
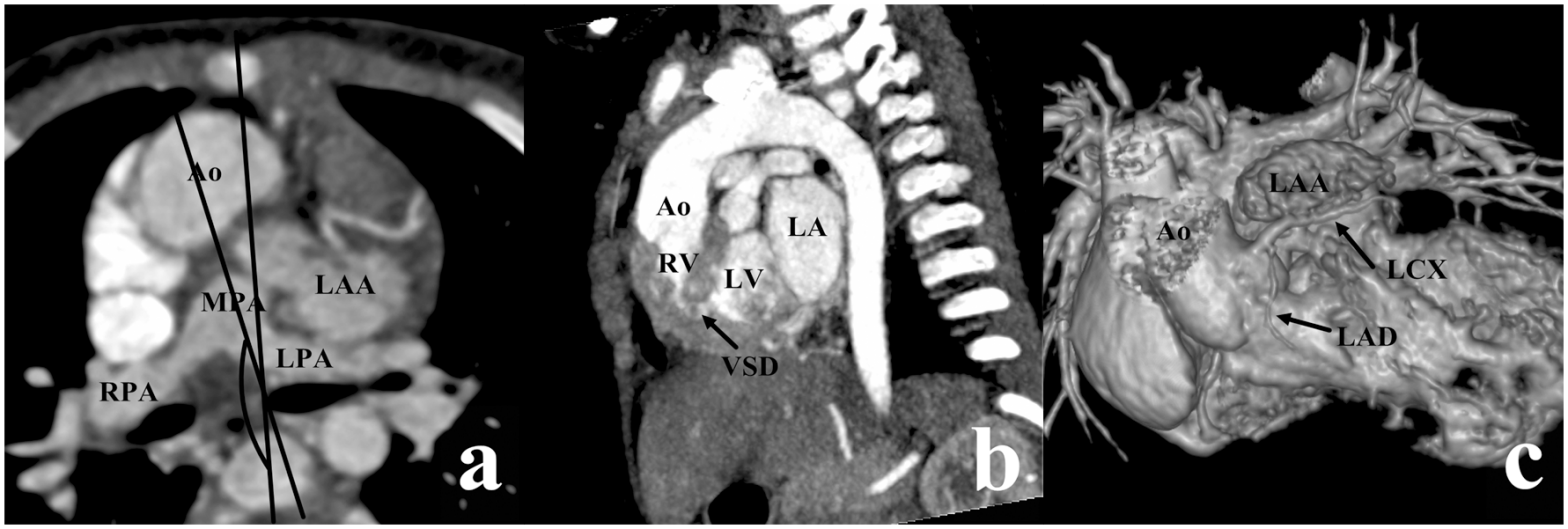
DORV with noncommitted VSD in a female aged 7 years. (a) The aorta (Ao) was anterior and to the right of the main pulmonary artery (MPA) (the measured angle = 167°). (b) The VSD (arrow) is far from both the two arterial valves. (c) The volume rendering (VP) image showed the dysplasia of the left anterior descending artery (LAD) and anomalous course of the left circumflex artery (LCX) crossing anterior of the left auricular appendage (LAA). LA, left atrium; LPA, left pulmonary artery; LV, left ventricle; MPA, main pulmonary artery; RPA, right pulmonary artery; RV, right ventricle.

According to Lev et al., there are four types of VSD, depending on the location of VSD relative to the two arterial valves [9]. If VSD was close to the aortic valve with its superior border lower than the inferior border of the aortic valve, it was termed a subaortic VSD (Figs 1b and 2b). In a similar manner, if VSD was close to the pulmonary valve with its superior border lower than the inferior border of the pulmonary valve, it was termed a subpulmonary VSD (Fig 3b), and if VSD was under both the aortic and pulmonary valves with equal distance, it was termed a double-committed VSD (Fig 4b). In a subset of patients, the distance between VSD and the two arterial valves was greater than the diameter of the Ao, which was termed a noncommitted VSD (Fig 5b) [10, 11]. The alignment of the two great arteries was determined by an angle between two specific lines. One was delineated to connect the sternum and center of the thoracic vertebral body and the other connected the two great arteries [11]. The measured angle increased as the line between the two great arteries rotated clockwise. The above measurement was performed on the transverse view through pulmonary bifurcation. If the Ao was posterior and to the right of the main pulmonary artery (MPA), in other words, 0° ≤ the measured angle < 90°, it was termed the RP position (Fig 1a); if two great arteries were side-by-side, in other words, the measured angle was equal to 90° or 270°, it was termed the side-by-side position (Fig 4a); if the Ao was anterior and to the right of the MPA (90° < the measured angle ≤ 180°), it was termed the RA position (Figs 3a and 5a); if the Ao was anterior and to the left of the MPA (180° < the measured angle < 270°), it was termed the LA position (Fig 2a).

In addition, to compare the diagnostic accuracies of DSCT with that of TTE, we categorized all the surgically confirmed malformations into four groups, i.e., intracardiac anomalies, coronary artery anomalies (CAAs), anomalies of great vessels, and separate thoracic and abdominal anomalies. Intracardiac anomalies included right ventricular outflow tract stenosis, pulmonary valve stenosis, atrial septal defect, tricuspid atresia, and bicuspid pulmonary valve. Anomalies of great vessels included pulmonary artery anomalies, persistent left superior vena cava, anomalous pulmonary venous connection, patent ductus arteriosus, right aortic arch, interrupted aortic arch, and coarctation of the aorta. Separate thoracic and abdominal anomalies referred to situs inversus and aortopulmonary collateral vessels.

### Assessment of image quality

The overall image quality of retrospective ECG-gated DSCT was evaluated by the above-mentioned two radiologists using a four-point scale in which 4 = excellent (excellent image quality, excellent anatomic details), 3 = good (good image quality, clear visualization of anatomic details), 2 = fair (fair image quality, anatomic structures required clinically could be identified), and 1 = poor (poor image quality, severe artifacts that incomplete or no useful anatomic information obtained). A score of 3 or 4 was considered to be diagnostic. Differences in opinions were resolved by discussion to reach a consensus.

### Trans-thoracic echocardiography

All patients underwent TTE preoperatively using a Philips SONOS 7500 ultrasound system (Philips Medical Systems, Bothell, WA). The examinations were performed on the basis of recommendations of the America Society of Echocardiography Committee [12]. An experienced echocardiographic investigator who was not involved in the DSCT diagnostics interpreted the TTE in a blind fashion.

### Radiation dose estimation

Radiation dose parameters, including volume CT dose index (CTDI_vol_) and dose-length product (DLP) were automatically displayed on the CT console after the examination. Infant-specific DLP conversion coefficients, based on the 2007 recommendations of the International Commission on Radiological Protection, were used to calculate the effective dose (ED) using conversion coefficients of 0.039 for patients <4 months of age, 0.026 for patients between 4 months and 1 year of age, 0.018 for patients between 1 and 6 years of age, and 0.012 for patients between 6 and 10 years of age [6, 13, 14].

### Statistical analysis

The assembled data were analyzed using the SPSS software for Windows (version 19.0, SPSS Inc., Chicago, IL, USA). The sensitivity, specificity, positive predictive value, and negative predictive value of DSCT and TTE were evaluated for intracardiac anomalies, CAAs, anomalies of great vessels, and separate thoracic and abdominal anomalies groups. Continuous variables were presented as means ± standards deviations. Categorical variables were expressed as numbers and percentages. Agreement of image quality and diagnosis of associated malformations between DSCT and TTE were assessed using the kappa value. Kappa values of 0.61–0.80 were considered to indicate good agreement and 0.81–1.00 were considered to indicate excellent agreement.

## Results

### Assessment of image quality

DSCT scanning were successfully performed in all the 47 pediatric patients. After consensus, the mean image quality score of 47 cases was 3.70 ± 0.46. All the DSCT images were considered as diagnostic, including 70.2% (33/47) were scores of 4, and 29.8% (14/47) were scores of 3. The interobserver agreement of image quality was calculated as κ = 0.76, which indicated good agreement.

### Associated multiple malformations

Apart from DORV, there were a total of 109 associated malformations confirmed by surgical results. A summary of the findings obtained with DSCT and TTE are shown in Table 1. DSCT missed detecting one bicuspid pulmonary valve (1/8) and three atrial septal defects (3/9) in addition to misdiagnosing one atrial septal defect (1/38) and one bicuspid pulmonary valve (1/39). TTE missed detecting one pulmonary artery anomaly (1/11), one aortopulmonary collateral vessel (1/10), one anomalous pulmonary venous connection (1/6), one interrupted aortic arch (1/2), one coarctation of the aorta (1/5), two cases of situs inversus (2/3), and three CAAs (3/15) in addition to misdiagnosing one pulmonary artery anomaly (1/36). The agreement of diagnosis of associated malformations between the two methods was calculated as κ = 0.90, which indicated excellent agreement. The sensitivity, specificity, positive predictive value, and negative predictive value of DSCT and TTE for each group are presented in Table 2.

**Table 1:** A summary of the findings obtained with DSCT and TTE (n = 47).

Associated malformations	Surgical results	DSCT findings				TTE findings			
		TP	FN	TN	FP	TP	FN	TN	FP
Right ventricular outflow tract stenosis	18	18	0	29	0	18	0	29	0
Pulmonary valve stenosis	8	8	0	39	0	8	0	39	0
Pulmonary artery anomalies	11	11	0	36	0	10	1	35	1
Aortopulmonary collateral vessels	10	10	0	37	0	9	1	37	0
Persistent left superior vena cava	5	5	0	42	0	5	0	42	0
Coronary artery anomalies	15	15	0	32	0	12	3	32	0
Anomalous pulmonary venous connection	6	6	0	41	0	5	1	41	0
Atrial septal defect	9	6	3	37	1	9	0	38	0
Patent ductus arteriosus	2	2	0	45	0	2	0	45	0
Right aortic arch	4	4	0	43	0	4	0	43	0
Interrupted aortic arch	2	2	0	45	0	1	1	45	0
Coarctation of the aorta	5	5	0	42	0	4	1	42	0
Tricuspid atresia	3	3	0	44	0	3	0	44	0
Situs inversus	3	3	0	44	0	1	2	44	0
Bicuspid pulmonary valve	8	7	1	38	1	8	0	39	0
Total	109	105	4	594	2	99	10	595	1

TP, true positive finding; FN, false negative finding; TN, true negative finding; FP, false positive finding.

**Table 2:** The diagnostic accuracies of DSCT and TTE according to anomalies categories.

Anomalies categories	DSCT				TTE			
	Sen	Spec	PPV	NPV	Sen	Spec	PPV	NPV
Intra-cardiac anomalies	91.30%	98.94%	95.45%	97.91%	100%	100%	100%	100%
CAAs	100%	100%	100%	100%	80.00%	100%	100%	91.43%
Anomalies of great vessels	100%	100%	100%	100%	88.57%	99.66%	96.87%	98.65%
Separate thoracic and abdominal anomalies	100%	100%	100%	100%	76.92%	100%	100%	96.43%

Sen, sensitivity; Spec, specificity; PPV, positive predictive value; NPV, negative predictive value; CAAs, coronary artery anomalies.

### Location of VSD and alignment of the two great arteries

The locations of VSD and the alignments of the two great arteries are listed in Table 3. Among the 47 cases, subaortic VSD was observed in 57.4% (27/47) of the cases, subpulmonary VSD was observed in 23.4% (11/47), double-committed VSD was observed in 14.9% (7/47), and noncommitted VSD was observed in 4.2% (2/47).

**Table 3:** The locations of VSD and the alignments of the two great arteries.

	Subaortic VSD	Subpulmonary VSD	Double-committed VSD	Noncommitted VSD	Total
RP	12 (75.0%)	3 (18.7%)	1 (6.3%)	0	16
Side-by-side	4 (33.3%)	2 (16.7%)	5 (41.7%)	1 (8.3%)	12
RA	6 (46.2%)	5 (38.4%)	1 (7.7%)	1 (7.7%)	13
LA	5 (83.3%)	1 (16.7%)	0	0	6
Total	27	11	7	2	47

RP, the aorta (Ao) was posterior and to the right of the main pulmonary artery (MPA); RA, the Ao was anterior and to the left of the MPA; LA, the Ao was anterior and to the right of the MPA.

The results for the alignment of the two great arteries showed that 34.0% (16/47) of the alignments were classified as the RP position, of which 75.0% (12/16) were subaortic VSD; 25.5% (12/47) were classified as the side-by-side position, of which 41.7% (5/12) and 33.3% (4/12) were double-committed VSD and subaortic VSD, respectively; 27.6% (13/47) were classified as the RA position, of which 46.2% (6/13) and 38.4% (5/13) were subaortic VSD and subpulmonary VSD, respectively; and 12.7% (6/47) were classified as the LA position, of which 83.3% (5/6) was subaortic VSD.

### Radiation dose estimation

As shown in Table 4, the mean DLP for patients <4 months of age was 31.11 ± 12.42 mGy·cm, which corresponds to an estimated mean ED of 1.21 ± 0.48 mSv. The mean DLP for patients between 4 months and 1 year of age was 40.77 ± 6.53 mGy·cm, which corresponds to an estimated mean ED of 1.06 ± 0.17 mSv. The mean DLP for patients between 1 and 6 years of age was 50.13 ± 2.73 mGy·cm, which corresponds to an estimated mean ED of 0.90 ± 0.18 mSv. The mean DLP for patients between 6 and 10 years of age was 46.28 ± 13.00 mGy·cm, which corresponds to an estimated mean ED of 0.55 ± 0.15 mSv. The estimated mean effective dose was 0.88 ± 0.34 mSv (<1 mSv).

**Table 4:** Radiation dose estimation according to different age groups.

	< 4 months	4 months to 1 years	1 years to 6 years	6 years to 10 years
CTDI_vol_ (mGy)	4.33 ± 2.56	10.79 ± 3.33	12.55 ± 4.53	8.64 ± 4.59
DLP (mGy·cm)	31.11 ± 12.42	40.77 ± 6.53	50.13 ± 2.73	46.28 ± 13.00
ED (mSv)	1.21 ± 0.48	1.06 ± 0.17	0.90 ± 0.18	0.55 ± 0.15

CTDI_vol_, volume CT dose index; DLP, dose-length product; ED, effective dose.

## Discussion

With high temporal resolution and fast scanning speed, DSCT has been widely used for pediatric cardiac examinations in recent years. However, the pediatric population is sensitive to ionizing radiation, and radiation exposure will increase their potential cancer risk. Li et al. reported that there was a negative correlation between radiation effect and patient age [15]. Therefore, minimizing the delivered radiation dose is necessary whenever possible, particularly in pediatric patients.

In this study, scanning was performed using a retrospective ECG-gated protocol rather than a prospective ECG-gated protocol. Although the radiation dose of the prospective ECG-gated protocol was lower than that of the retrospective ECG-gated protocol [8, 16], the former is characterized by acquiring data from a programmed triggering point in every cardiac cycle, which relies on low and stable heart rhythm for diagnostic image quality [5, 17]. Thus, in cases with relatively high or unstable heart rhythm, the achieved image quality, particularly of intracardiac structures and coronary arteries, is unsatisfactory because the start point of data acquisition is not compatible with the cardiac cycle [6, 8]. In contrast, the retrospective ECG-gated protocol has the ability to perform ECG editing, which is a technique that regulates the position of the ECG-pulsing window within the cardiac cycle to adapt to the relatively high or irregular heart rhythm [18]. Then, the acquired data will be retrospectively selected for image reconstruction. Thus, with respect to pediatric patients with relatively high or unstable heart rhythm, these procedures can provide high-quality images with minimal artifacts, particularly of intracardiac details and coronary arteries.

How to reduce radiation dose is the most important issue when undergoing cardiac CT because young patients are more radiosensitive than adults [19]. We took several measures to reduce radiation dose. First, tube voltage is one of the major factors that affect radiation exposure. In this study, the tube voltage was set at a low level of 80 kV. Decreasing the tube voltage will not merely decrease radiation exposure. Actually, for cardiac CT, low tube voltage not only achieves higher vascular enhancement without the loss of contrast-to-noise ratio but also reduces the contrast agent dose [20, 21]. Second, data were acquired using an ECG-based tube current modulation technique. That means that during the prescribed R–R interval (ECG-pulsing window), the tube current was preset at full dose, whereas in the remainder of the R–R interval, the tube current was reduced to a low level of 4%–20% of the full dose, which dramatically reduced the radiation exposure [22]. Third, the heart rhythm adaptive pitch, a technique that allows for an increase in pitch to fit a high heart rhythm, was applied. According to McCollough et al., increasing the pitch from 0.20 to 0.46 will decrease the CTDI_vol_ by nearly 57% [23]. Therefore, the heart rhythm adaptive pitch will further reduce radiation exposure and shorten the duration of examination, particularly in pediatric patients with elevated or irregular heart rhythm who cannot hold their breath well. In summary, the estimated mean ED was <1 mSv (0.88 ± 0.34 mSv) because the above-mentioned measures were applied, which was acceptable for pediatric patients.

According to the data presented in Table 2, DSCT appears to be slightly inferior to TTE for the detection of intracardiac anomalies (sensitivity: DSCT, 91.30%; TTE, 100%). DSCT missed several tiny anomalies such as atrial septal defect (3/9) and bicuspid pulmonary valve (1/8). The reason is that DSCT imaging is a digital-based technology that requires a workstation to convert digital information to gray-scale images; thus, some intracardiac details are too tiny to demonstrate with certainty. Under this circumstance, we could not make an exact decision at times on whether the anomalies existed or not, which means that there was a possibility of missed diagnoses. However, dynamic functional sequels and color Doppler flow imaging are unique advantages of TTE that enable the detection of such tiny anomalies and enable dynamic assessments of intracardiac structures and cardiac functions [24]. In the recent years, however, several studies have shown that retrospective ECG-gated DSCT is feasible for evaluating the dynamic characteristics of the heart [25, 26], but TTE appears to be more flexible and convenient.

CAAs are usually associated with complex CHD. Yu et al. reported that the prevalence of CCAs in DORV was approximately 21.43% [27]. Our study had 15 cases (15/47, 31.91%) with CAAs. Therefore, the visualization of the origin and distribution of the anomalous coronary arteries preoperatively is critical for reducing the risk of surgery. Table 2 indicates that DSCT was more suitable than was TTE for the detection of coronary arteries (sensitivity: DSCT, 100%; TTE, 80.00%) because of its powerful data postprocessing techniques such as MPR, MIP, and VP. The limitation of TTE in providing three-dimensional information impaired its ability for the successful diagnosis of CAAs by increasing the rate of false negatives (DSCT, 0%; TTE, 25.00%) [28].

In addition, DSCT was superior to TTE in detecting anomalies of great vessels and separate thoracic and abdominal anomalies (sensitivity, anomalies of great vessels: DSCT, 100%; TTE, 88.57%; separate thoracic and abdominal anomalies: DSCT, 100%; TTE, 76.92%). DSCT can visualize noncardiac anatomical details and gives a wide field of view of the thorax and abdomen. Nevertheless, the diagnostic value of TTE, particularly for the distal segment of great vessels and separate abdominal anomalies, is restricting because of its limited acoustic window [24, 29]. Furthermore, the experience of the echocardiographic investigator should not be neglected as well.

Our study suggested that retrospective ECG-gated DSCT has satisfactory diagnostic accuracy in the preoperative assessment of DORV and associated multiple malformations in pediatric patients and can provide excellent diagnostic image quality without the disadvantage of increasing the radiation dose. However, it has the possibility of missed diagnoses for tiny intracardiac malformations. So combining the results of DSCT and TTE would prove beneficial to guarantee the diagnostic accuracy of DSCT in these malformations.

### Limitations

We need to acknowledge several limitations of this study. First, we included a relatively small number of patients, which limits us to extend our results to a large group. Second, although retrospective ECG-gated DSCT was an accurate modality demonstrating intra- and extracardiac malformations, however, it had the possibility of missed diagnoses for tiny intracardiac malformations such as atrial septal defect due to its image resolution limitation. Hence, we should combine the results of the two modalities if DSCT could not determine those malformations. Third, like TTE, retrospective ECG-gated DSCT is capable of analyzing cardiac function and providing hemodynamic information. We could not provide the functional information, especially the right ventricular function, which was helpful in the assessment of DORV. We expect that in the future, more studies will focus on the assessment of cardiac function in DORV patients by using retrospective ECG-gated DSCT.

In summary, retrospective ECG-gated DSCT is a better diagnostic tool than TTE for pediatric patients with complex congenital heart disease such as DORV. Combined with TTE, it may reduce or even obviate the use of invasive cardiac catheterization, and thus exposing the patients in a much lower radiation dose.
